# First report of prevalence and assemblage analysis of *Giardia duodenalis* in pigs from Guangxi Zhuang Autonomous Region, southern China[Fn FN1]

**DOI:** 10.1051/parasite/2023051

**Published:** 2023-11-28

**Authors:** Ya-Fei Song, Meng-Jie Chu, Fei Huang, Yang Liu, Hui-Hong Lu, Si-Ang Li, Shu-Yan Wang

**Affiliations:** 1 Guangxi Vocational University of Agriculture Nanning 530007 China; 2 Key Laboratory of Fujian-Taiwan Animal Pathogen Biology, College of Animal Sciences (College of Bee Science), Fujian Agriculture and Forestry University Fuzhou 350002 China

**Keywords:** *Giardia duodenalis*, Pigs, Prevalence, Multilocus genotyping, Guangxi Zhuang Autonomous Region

## Abstract

*Giardia duodenalis* is a common intestinal protozoan that can cause diarrhea and intestinal disease in animals and in humans. However, the prevalence and assemblages of *G. duodenalis* in pigs from Guangxi Zhuang Autonomous Region have not been reported. In this study, a total of 724 fecal samples (201 from nursery pigs, 183 from piglets, 175 from breeding pigs, and 165 from fattening pigs) were obtained in four areas of the region (Nanning, Yulin, Hezhou, and Guigang). The gene of the small subunit ribosomal RNA (*SSU rRNA*) of *G. duodenalis* was amplified by nested PCR. The results show that the prevalence of *G. duodenalis* in pigs was 3.59% (26/724), of which 14 samples belonged to assemblage A (53.85%) and 12 samples belonged to assemblage E (46.15%). The infection rates of *G. duodenalis* in Hezhou, Yulin, Nanning, and Guigang were 0%, 0.7%, 10.8% and 1.1%, respectively (*χ*^2^ = 45.616, *p* < 0.01); whereas 5.1% of breeding pigs, 6.0% of piglets, 2.4% of fattening pigs, and 1.0% of nursery pigs were infected with *G. duodenalis* (*χ*^2^ = 8.874, *p* < 0.05)*.* The *SSU rRNA*-positive samples were amplified by PCR based on the β-giardin (*bg*), glutamate dehydrogenase (*gdh*), and triphosphate isomerase (*tpi*) genes. Ten, eight and seven positive samples were detected, respectively. Based on phylogenetic analysis of the three genetic loci sequences, a multilocus genotyping A1 was found. The findings of this study provide basic data for the development of prevention and control of *G. duodenalis* infections in pigs and humans in the Guangxi Zhuang Autonomous Region.

## Abstract (fr)

*Giardia duodenalis* est un protozoaire intestinal commun qui peut provoquer des diarrhées et des maladies intestinales chez les animaux et les humains. Cependant, la prévalence et les assemblages de *G. duodenalis* chez les porcs de la région autonome Zhuang du Guangxi n’ont pas été rapportés. Dans cette étude, un total de 724 échantillons fécaux (201 provenant de jeunes porcelets, 183 de porcelets, 175 de porcs reproducteurs et 165 de porcs à l’engrais) ont été obtenus dans quatre zones de la région (Nanning, Yulin, Hezhou et Guigang). Le gène de la petite sous-unité de l’ARN ribosomal (ARNr SSU) de *G. duodenalis* a été amplifié par PCR nichée. Les résultats ont montré que la prévalence de *G. duodenalis* chez les porcs était de 3,59 % (26/724), dont 14 échantillons appartenaient à l’assemblages A (53,85 %) et 12 échantillons à l’assemblage E (46,15 %). Les taux d’infection par *G. duodenalis* à Hezhou, Yulin, Nanning et Guigang étaient respectivement de 0, 0,7 %, 10,8 % et 1,1 % (χ^2^ = 45,616, p < 0,01), alors que 5,1 % des porcs reproducteurs, 6,0 % des porcelets, 2,4 % de porcs à l’engrais et 1,0 % des jeunes porcelets étaient infectés par *G. duodenalis* (χ^2^ = 8,874, p < 0,05). Les échantillons positifs pour l’ARNr SSU ont été amplifiés par PCR basée sur les gènes de la β-giardine (bg), de la glutamate déshydrogénase (gdh) et de la triphosphate isomérase (tpi), et dix, huit et sept échantillons positifs ont été détectés, respectivement. Sur la base de l’analyse phylogénétique des trois séquences de loci génétiques, un génotypage multilocus A1 a été trouvé. Les résultats de cette étude fournissent des données de base pour le développement de la prévention et du contrôle des infections à *G. duodenalis* chez les porcs et les humains dans la région autonome Zhuang du Guangxi.

## Introduction

*Giardia* spp*.* are common intestinal parasites, affecting both humans and a wide variety of other animals [13]. The parasite was first discovered over 300 years ago by Antonie van Leeuwenhoek, and since then, six distinct *Giardia* species have been described [9]. The six described *Giardia* species (*Giardia agilis*, *Giardia psittaci, Giardia ardeae*, *Giardia muris*, *Giardia microti*, and *Giardia duodenalis*) infect a wide range of animals including birds, amphibians, rodents, and mammals [20]. *Giardia duodenalis* is an important zoonotic parasite that can cause giardiasis [32, 35]. The life cycle of *G. duodenalis* is simple. It consists of two key stages: rapidly multiplying trophozoites which attach to intestinal epithelial cells, and cysts with high resistance to environmental degradation, that are released in the feces and spread through the fecal-oral route [11, 19, 23]. Infection with *G. duodenalis* shows a wide range of clinical symptoms, such as acute or chronic diarrhea, nausea, abdominal pain, vomiting, and weight loss [10, 39]. Furthermore, giardiasis affects growth, development, and cognitive functions in infected children [13, 34].

The comprehension of *G. duodenalis* through molecular biological analysis has greatly contributed to the understanding of its taxonomy, population genetics, and epidemiology. The small subunit ribosomal RNA (*SSU rRNA*), β-giardin (*bg*), glutamate dehydrogenase (*gdh*), and triose phosphate isomerase (*tpi*) genes are four commonly used gene loci in the genotyping of *G. duodenalis*, but a single gene may not correctly identify *G. duodenalis* or completely describe its genetic characterization [25, 43]. Multilocus genotyping (MLG) based on more than three genes is considered to be more reliable than single-locus genotyping in assemblage and sub-assemblage typing of isolates. As a result, it has been widely used to investigate the genotypic diversity of *G. duodenalis*, and is considered to be useful for detecting and identifying mixed infections by different assemblages of the parasite [13, 37]. To date, *G. duodenalis* can be divided into eight assemblages (A–H) based on genetic analysis, with each assemblage exhibiting a distinct host range [5, 20, 27]. Among the assemblages, assemblages A and B can be found in a wide array of mammals, including humans and pigs [13, 31]. Other assemblages (C–H) exhibit host specificity and narrow host ranges: assemblages C and D are specific to dogs and other canids; assemblage E is found in domestic animals, such as pigs, cattle, and horses; assemblage F has been identified in cats; assemblages G and H are found in rodents and marine animals such as seals, respectively [8, 13, 26]. However, assemblages C–F have also been identified in humans [22, 29, 35, 44]. These findings suggest that close contact between humans and animals may lead to human infection with *G. duodenalis*.

Although some studies have reported prevalence and distribution of *G. duodenalis* in pigs [1, 15, 17, 18, 41], there are no reports on the infection of *G. duodenalis* in pigs in Guangxi Zhuang Autonomous Region, China. Pigs infected with *G. duodenalis* may cause malabsorption and weight loss, resulting in a decline in pig production [48]. Currently, assemblages A–F have been found in pigs, with assemblage E the preponderant genotype [4]. Therefore, this study aimed to investigate the prevalence of *G. duodenalis* infection and identify the genotypes present in pigs from Guangxi Zhuang Autonomous Region, China. The study provides essential data concerning *G. duodenalis* infections in pigs in southern China, which can contribute to the development of targeted public health and effective strategies for prevention and control of giardiasis in this area.

## Materials and methods

### Samples collection

**Figure 1:**
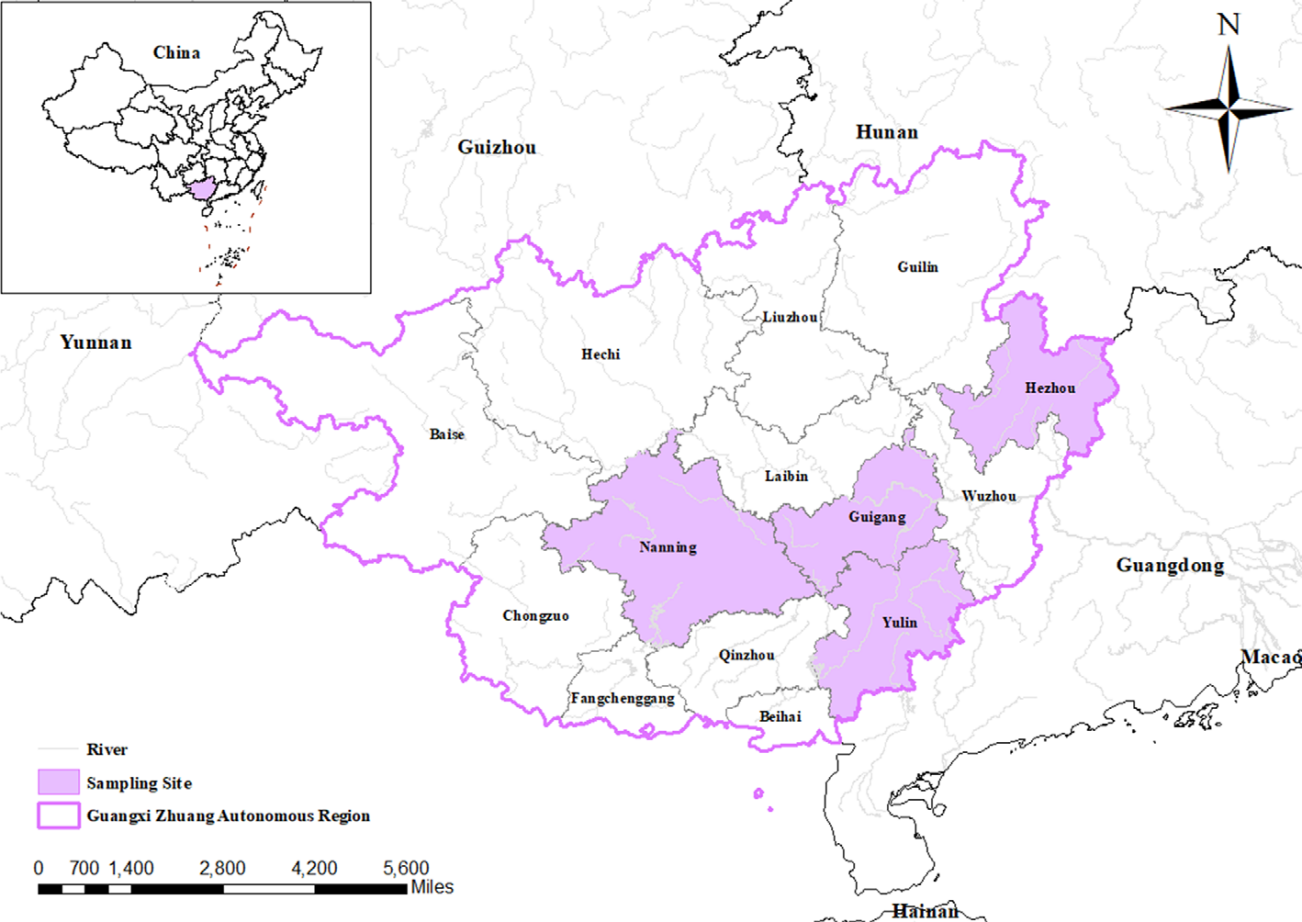
Map of the sample collection area. The green areas are the four areas where samples were collected in Guangxi Zhuang Autonomous Region, China.

From March 2021 to May 2022, a total of 724 fecal samples (201 from nursery pigs, 183 from piglets, 175 from breeding pigs, and 165 from fattening pigs) were collected from four cities (Nanning, Yulin, Hezhou, and Guigang) in Guangxi Zhuang Autonomous Region, China (Fig. 1). Fecal samples were collected directly from the rectum by using sterile gloves. Subsequently, fecal samples were placed in sterile plastic bags marked with the date, type and farm. All samples were promptly transported to the laboratory in cool boxes with ice packs and stored at −80 °C until DNA extraction.

### Genomic DNA extraction

Approximately 200 mg of each fecal sample was aseptically transferred to individual 1.5 mL centrifuge tubes. Genomic DNA extraction was performed using an E.Z.N.A.^®^ Stool DNA Kit (D4015-02, OMEGA Bio-Tek Inc., Norcross, GA, USA), according to the manufacturer’s instructions. The extracted DNA was subsequently stored at −20 °C to maintain its integrity for subsequent analyses.

### PCR amplification

All samples were amplified by using nested PCR targeting the *SSU rRNA* gene, as previously described [2]. The PCR reaction mixture comprising 25 μL was prepared and amplified according to the procedure described by Jing *et al.* [15]. All *SSU rRNA*-positive samples were subjected to amplification using nested PCR targeting the *bg*, *gdh*, and *tpi* genes, as previously described [7, 16, 36]. A positive control (DNA from *G. duodenalis* stored in the laboratory at −80 °C) and a negative control (distilled water) were included in each PCR assay. Each sample was processed three times at the *SSU rRNA* gene and each *SSU rRNA*-positive sample was processed three times at the *bg*, *gdh*, and *tpi* genes. The PCR products were identified by 1.5% agarose gel electrophoresis (Gene Biotechnology International Trade Co., Ltd., Shanghai, China).

### Sequencing and phylogenetic analysis

PCR products of positive samples were processed for two-directional sequencing by Sangon Biotech (Xiamen, China). The obtained DNA sequences were aligned with homologous sequences available in the GenBank database of the National Center for Biotechnology Information (https://www.ncbi.nlm.nih.gov/). The MLG method was only used to analyze the genetic diversity of positive samples which were successfully sequenced at all three gene loci (*bg*, *gdh*, and *tpi*). Neighbor-joining trees [33] were constructed using Mega 11 software, employing the kimura-2 parameter model. The reliability of these trees was assessed using the bootstrap method with 1,000 pseudoreplicates.

### Statistical analysis

The Chi-square test (SPSS 25.0 Inc., Chicago, IL, USA) was used to assess differences in prevalence between regions, feeding stages and farming practices. Differences of *p* < 0.05 were considered statistically significant, and differences of *p* < 0.01 were considered extremely significant.

### Nucleotide sequence accession numbers

All nucleotide sequences have been submitted to the GenBank database in NCBI and allocated accession numbers as follows: OQ943958–OQ943959 for the *SSU rRNA* gene, OQ934094–OQ934095 for the *bg* gene, OQ934096–OQ934101 for the *gdh* gene, and OQ934102–OQ934104 for the *tpi* gene.

## Results

Out of 724 fecal samples collected from 4 cities, 26 samples (26/724) tested positive for *G. duodenalis* based on the *SSU rRNA* gene. The highest infection rate of *G. duodenalis* was in Nanning (10.8%, 23/213), followed by Guigang (1.1%, 2/177), Yulin (0.7%, 1/146), and Hezhou (0%, 0/188). Statistical analysis demonstrated significant variations in the positive rate of *G. duodenalis* infection with pigs among different regions (*χ*^2^ = 45.616, *p* < 0.01). Among the various feeding stages, piglets had the highest infection rate (6%, 11/183), followed by breeding pigs (5.1%, 9/175), fattening pigs (2.4%, 4/165), and nursery pigs (1%, 2/201) (*χ*^2^ = 8.874, *p* < 0.05). The prevalence of *G. duodenalis* infection in intensive and free-range farms were 3.7% (15/410) and 3.5% (11/314), respectively. Statistical analysis showed no significant difference between the two groups (*p* > 0.05) (Table 1).

**Table 1:** Investigation of *G. duodenalis* infection in pigs in Guangxi Zhuang Autonomous Region.

Category	No. of specimens	No. of positive (%)	*SSU rRNA* (*n*)	Genotype			*p*
				*bg* (*n*)	*gdh* (*n*)	*tpi* (*n*)	
Regions							<0.01
Hezhou	188	0					
Yulin	146	1 (0.7)	E (1)		E (1)		
Nanning	213	23 (10.8)	A (13), E (10)	A (5), E (4)	A (6), E (1)	A (4), E (1)	
Guigang	177	2 (1.1)	A (1), E (1)	A (1)		A (1), E (1)	
Feeding stages							<0.05
Breeding pigs	175	9 (5.1)	A (9)	A (4)	A (5)	A (4)	
Piglets	183	11 (6)	A (1), E (10)	A (1), E (4)	E (1)	E (2)	
Fattening pigs	165	4 (2.4)	A (4)	A (1)	A (1)		
Nursery pigs	201	2 (1)	E (2)		E (1)	E (1)	
Farming methods							>0.05
Intensive farming	410	15 (3.7)	A (5), E (10)	A (2), E (4)	A (1), E (1)	A (1), E (1)	
Free-range	314	11 (3.5)	A (9), E (2)	A (4)	A (5), E (1)	A (4), E (1)	
Total	724	26 (3.6)	A (14), E (12)	A (6), E (4)	A (6), E (2)	A (5), E (2)	

Sequence analysis of the *SSU rRNA* gene showed two assemblages (A and E) of *G. duodenalis* in pigs. Of the 26 *SSU rRNA*-positive samples, 14 isolates (53.85%, 14/26) belong to zoonotic assemblage A, while the other 12 isolates (46.15%, 12/26) were identified as assemblage E (Table 1). Ten, eight and seven sequences were obtained by amplifying 26 *SSU rRNA*-positive samples at the *bg*, *gdh*, and *tpi* gene loci, respectively (Table 1). At the *bg* locus, six and four isolates belong to assemblages A and E, respectively; at the *gdh* locus, six and two isolates belonged to assemblages A and E, respectively; and at the *tpi* locus, five and two isolates belonged to assemblages A and E, respectively (Table 1). Notably, only one fecal sample of assemblage A was successfully sequenced at all three gene loci and one MLG A1 was formed (Table 2).

**Table 2:** Genotyping of *G. duodenalis* based on the *SSU rRNA*, *bg*, *gdh*, and *tpi* genes.

Isolate	*SSU rRNA*	Genotype			MLG type
		*bg*	*gdh*	*tpi*	
YL-B14	E	–	E	–	
NN-2 (10)	A	A	A	–	
NN-5	A	A1	A1	A1	MLG A1
NN-7 (GG-40)	A	A	–	A	
NN-12	A	–	–	A	
NN-19	A	–	A	–	
NN-24	A	–	A	A	
NN-31 (GG-22)	E	–	–	E	
NN-32 (33, 38, 54)	E	E	–	–	
NN-46	E	–	E	–	
NN-81	A	A	–	–	
NN-86	A	–	A	–	
NN-11 (23, 83, 87)	A	–	–	–	
NN-34 (41, 43, 44)	E	–	–	–	

**Figure 2:**
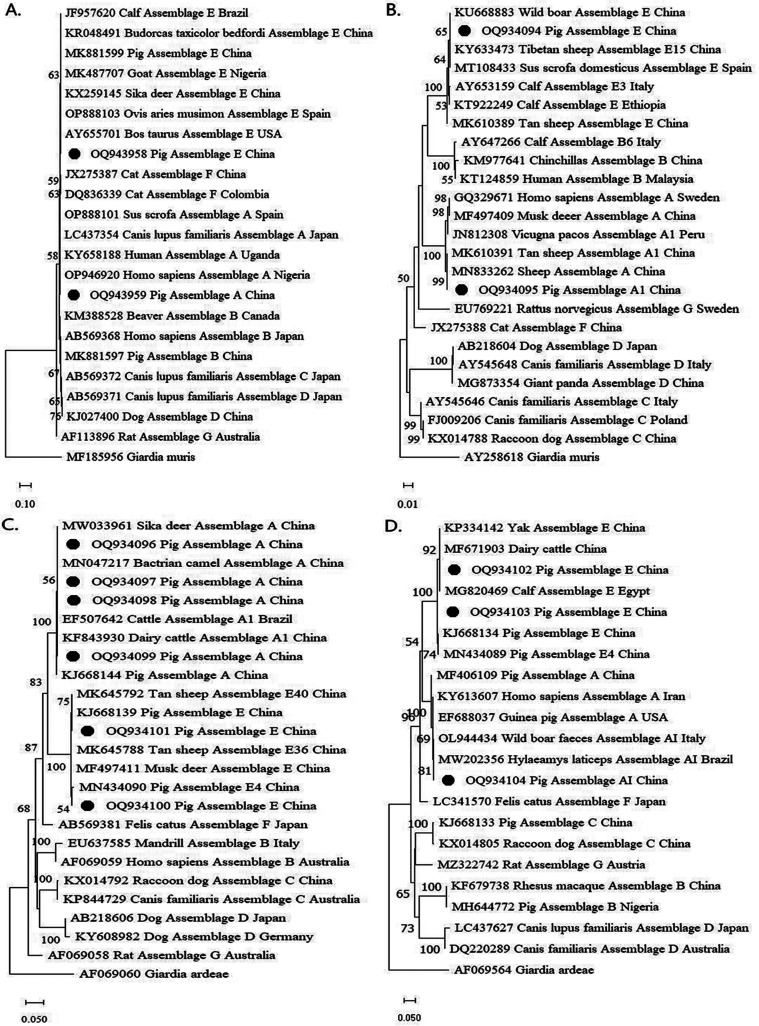
The phylogenetic relationships of *G. duodenalis* isolates were obtained by neighbor-joining analysis. Bootstrap values > 50% from 1,000 replicates are shown as nodes. The black circles represent the sequences obtained in this study. A. Phylogenetic relationships based on *SSU rRNA* nucleotide sequences; B. Phylogenetic relationships based on *bg* nucleotide sequences; C. Phylogenetic relationships based on *gdh* nucleotide sequences; D. Phylogenetic relationships based on *tpi* nucleotide sequences.

To explore the genetic relationships of the *G. duodenalis* isolates from pigs, four phylogenetic trees were constructed using the *SSU rRNA*, *bg*, *gdh*, and *tpi* sequences of the parasite. The results show that the representative isolates at the four gene loci were distributed into assemblages A and E, respectively (Fig. 2).

## Discussion

*Giardia duodenalis* is an intestinal parasitic protozoan that has attracted much attention in recent years. Infection with *G. duodenalis* is widespread in pigs, affecting individuals of all age groups [24]. In this study, four areas of the Guangxi Zhuang Autonomous Region were investigated, and it was found that the positive rate of *G. duodenalis* was 3.59% in 724 samples. There have been no reports of pigs infected with *G. duodenalis* in Vietnam, a neighboring region of the region. However, a report by Verle *et al.* showed that the prevalence of *G. duodenalis* infection in humans from Vietnam was 3.2% [38]. Therefore, the results of this study can provide a reference for Vietnam, and further research is needed to determine whether the cause of human infection with *G. duodenalis* is related to pigs [38]. The infection rates of *G. duodenalis* in pigs vary around the world, such as 1% (6/633) in Canada, 3.4% (3/90) in Brazil, 14% (120/856) in Denmark, 14.8% (110/745) in Korea, 25.4% (53/209) in Nigeria, and 31.1% (90/289) in Western Australia [1, 3, 6, 12, 17, 28]. In China, the infection rate of *G. duodenalis* in this investigation was similar to the prevalence in Sichuan Province (3.1%, 11/357) [21]; it was higher than that detected in Hubei Province (0.97%, 8/826), Xinjiang (2.6%, 21/801) and Henan Province (1.7%, 15/897) [15, 18, 40], but lower than that reported in Guangdong Province (18.04%, 94/521), Fujian Province (26.9%, 195/725), Shaanxi Province (8.0%, 45/560), and Zhejiang Province (10.5%, 13/124) [41, 46–48]. These differences can be attributed to several factors, including sample conditions, feeding patterns, age groups, testing methods and seasons changes [4]. We speculate that the strict prevention and control measures implemented after the African swine fever outbreak in China may have resulted in lower infection rates in this study than the rates reported in other regions of China. Moreover, the increased frequency and intensity of disinfection have played a crucial role in reducing the transmission of pathogens.

The positive rate in different regions and for different feeding stages was significantly different (*χ*^2^ = 45.616, *p* < 0.01; *χ*^2^ = 8.874, *p* < 0.05). The highest prevalence (10.8%, 23/213) was observed in Nanning, and the lowest prevalence (0%, 0/188) in Hezhou. Among the different feeding stages, the infection rates of breeding pigs (5.1%, 9/175) and piglets (6.0%, 11/183) were higher than those of fattening pigs (2.4%, 4/165) and nursery pigs (1.0%, 2/201). Similarly, a study in Henan Province found that piglets had higher infection rates (5.8%, 14/243) compared to fattening pigs (0.2%, 1/439) [40]. In contrast, a study in Xinjiang found that the infection rate was highest in fattening pigs (5.4%, 7/129) and lowest in pre-weaned piglets (1.2%, 2/169) [15]. Previous studies have shown that different prevalence in pigs of different age groups may be caused by gut microbiota, nutritional status, immunity and geographical isolation [28]. The higher rate of infection in piglets may be attributed to reduced immunity. During this stage, piglets have not yet fully developed their own immune system [6, 24]. Additionally, factors such as environmental conditions, inadequate nutrition, and stress can further compromise their immune defenses. These situations increase susceptibility to *G. duodenalis* and other pathogens.

No significant differences were found in the positive rates for different feeding methods in our study (*p* > 0.05). The positive rate of intensive feeding farms was 3.7% (15/410), while the positive rate of free-range farms was 3.5% (11/314). The low positive rates of both intensive feeding and free-range farms indicates that people have begun to pay attention to the prevention and control of giardiasis. However, the possibility that insufficient samples on free-range farms led to lower infection rates cannot be ruled out.

*Giardia duodenalis* zoonotic assemblage A (*n* = 14, 53.85%) and assemblage E (*n* = 12, 46.15%) were identified among the 26 samples. Assemblage A was the predominant genotype in this study. However, in Australia, Armson *et al.* identified 56 *G. duodenalis* positive samples belonging to assemblage E (37 samples, 64.9%) and assemblage A (19 samples, 33.3%) [3]. A study in Denmark found that 13 *G. duodenalis* positive samples belonged to assemblage E (11 samples, 84.6%) and assemblage A (2 samples, 15.4%) [28]. The finding that pig-derived assemblage A isolates have 100% homology with human-derived isolates at the *SSU rRNA* locus means the possibility of zoonotic transmission in this areas. Notably, we also identified assemblage E, which has been reported to infect humans [14].

To further expand knowledge of the genetic diversity of *G. duodenalis* in pigs, the sequence characters of the *bg*, *gdh*, and *tpi* genes were analyzed for the 26 *SSU rRNA* positive samples and the MLGs were characterized in pigs based on data from these three loci. Amplification success rates at the *bg*, *gdh*, and *tpi* loci varied from 26.92% to 38.46%. The genetic loci of *Giardia* have different substitution rates, leading to different resolution for parasite typing at varied loci [13]. Nested PCR protocols based on multiple-copy genes (*e.g. SSU rRNA*) have higher diagnostic sensitivities than those based on single-copy genes (*bg*, *gdh*, *and tpi*) [45]. Therefore, the *SSU rRNA*-positive samples not amplified at the other three loci may be due to the limited sensitivity of PCR in detecting the single-copy genes. The present study confirmed that more positive samples of *G. duodenalis* can be amplified based on the *SSU rRNA* gene. The same problem has been reported in similar studies in pigs in Xinjiang [15]. The results showed that one fecal sample of assemblage A was successfully sequenced at all three loci, with one MLG, A1. In Ogun state of Nigeria, 12 *SSU rRNA*-positive samples were simultaneously amplified at three loci, with four MLGs [1]. In Shaanxi Province, eight *SSU rRNA*-positive samples were simultaneously amplified at three loci, with four MLGs [41]. In Fujian Province, six *SSU rRNA*-positive samples were simultaneously amplified at three loci, with one MLG [46]. In Spain, 76 *SSU rRNA*-positive samples from humans were simultaneously amplified at three loci, with 23 MLGs [42]. In Southwestern Iran, 82 *SSU rRNA*-positive samples from humans were simultaneously amplified at three loci, with two MLGs [30]. These results show that the polymorphism of *G. duodenalis* is different in different regions.

In short, pigs infected with *G. duodenalis* in Guangxi Zhuang Autonomous Region may be a potential source of infectious cysts that affect humans. Workers on pig farms and in slaughterhouses are at risk of infection with *G. duodenalis*. Understanding the transmission dynamics between animals and humans is crucial for effective disease control and prevention strategies. Therefore, public health problems caused by *G. duodenalis* should be investigated.

In conclusion, this study provides the first report of giardiasis in pigs from Guangxi Zhuang Autonomous Region, China. Although a low infection rate of *G. duodenalis* (3.59%, 26/724) was found in this study, the identification of assemblages A and E, and the predominance of assemblage A, suggest that pigs may play an important role in the transmission of *G. duodenalis*. The multilocus genotyping results of *bg*, *tpi*, and *gdh* loci showed only one MLG A1. The identification and understanding of the distribution of *G. duodenalis* in animals, such as pigs, are crucial for the development and implementation of effective prevention and control measures. These findings highlight the importance of continued surveillance and targeted interventions to mitigate the risk of *G. duodenalis* transmission between animals and humans. The data presented in this study serve as a foundation for future research on *G. duodenalis*.

## Conflict of interest

All authors declare that they have no conflict of interest.
